# Our New York Letter

**Published:** 1886-10

**Authors:** 


### OUR NEW YORK LETTER.

INTRA-PULMONARY INJECTIONS IN PHTHISIS—HOW IT IS PRACTICED IN CHARITY HOSPITAL—A REMARKABLE CASE OF FOREIGN BODY IN THE INTESTINES—INCREASED MORTALITY AMONG CHILDREN IN NEW YORK—ITS CAUSE, ETC.

*Editors Atlanta Medical and Surgical Journal:*

At a recent meeting of the Lenox Medical and Surgical Society, Dr. J. Blake White, visiting physician to Charity Hospital, read a paper on "Intra-pulmonary Injections in Phthisis." This procedure seemed to him so rational that he determined to give it a judicious trial during his service at the hospital. He believed the local application of alteratives was our best method for ridding the system of tubercular material, and that the most successful treatment for this disease would include every local and general means for checking the accumulation of tubercular exudation and arresting ulcerative processes. The walls of pulmonary cavities were compared to indolent ulcers, which require local treatment. The plan consisted in injecting a few minims of carbolized iodine through the chest-wall into the cavity by means of a modified hypodermic syringe. Under this treatment the most urgent symptoms were relieved, cough being controlled and expectoration diminished. A temporary paroxysm of coughing was common immediately after the injection. Ten patients were treated and all but three improved, which were very unfavorable cases. Dr. White believed this method promised good results, especially when the disease was not too far advanced.

He uses from m.x to m.xx of the following injection, which is slightly warmed:

℞. Tr. iodinii........................ʒ ii–ʒ iii.

Acid carbol........................m.xx.

Alcohol Dil. (50 per cent.)..............℥ ii.

M.

In the discussion following, the general opinion was that there is considerable difficulty in ascertaining the depth and exact locality of cavities, but the plan was considered a step forward in the management of a disease for which we have been able to do very little. Dr. White admitted these difficulties, but did not regard them insurmountable. As an adjuvant these injections were very useful, but climatic influences, nourishment and tonics were also necessary.

Since this paper was presented, Dr. White has reported the case of a female with a large phthisical cavity, who sweated profusely, was greatly prostrated and expectorated about 16 ounces daily. Under intra-pulmonary injections the expectoration has been reduced to less than ℥i. There is scarcely any cough, and to-day she looks well.

A rather remarkable case lately occurred at the Presbyterian Hospital during the service of Dr. Louis A. Stimson. The patient, an Italian, 30 years old, gave the following history:

About nine months previous to admission, he was seized with pains in the lower part of the abdomen on the left side, extending into the thigh. After being confined in bed for some time, he apparently recovered and seemed well up to a few days before admission, when he had pains in the left iliac fossa. An induration was felt here and the left thigh was partly flexed. Rectal examination was negative. Pain and more or less constitutional disturbance continued for about a month, when a fluctuating swelling appeared over the left glutial region. An exploratory puncture revealed blood, but no pus. Diarrhœa set in and the patient emaciated rapidly. The skin over the glutial tumor became red and thin and a spontaneous opening finally occurred, with the discharge of a large quantity of fœcal matter, blood clots and gas. A free incision was made into the tumor. Fœcal matter and blood could be seen oozing from the great sacro sciatic notch and the gluteous maximus muscle was partly gangrenous. Two days later the man died with heart failure. At the autopsy a lady's hat-pin, seven inches long and having a round glass head, was found lying in the splenic flexure of the colon, perforating the wall of the gut and resting against the ilium. In the sigmoid flexure were three small perforations, leading into an abscess cavity beneath the iliac fascia. The external iliac vein was eroded and opened. It is needless to say the diagnosis was not made during life, and how the hat-pin came there still remains a mystery.

For many years it has been the custom of the city Board of Health to appoint a summer corps of fifty physicians, whose duty it was, for the remuneration of $100 a month, to visit tenements and prescribe for children where parents were too poor to pay a doctor. The city was divided into fifty districts and a physician assigned to each. Tickets were distributed for the free excursions on the Floating Hospital of St. John's Guild and also for the Seaside Sanitarium. This year the Board of Apportionment, in the exercise of its economic functions, refused to appropriate the usual $10,000, and consequently there is no summer corps. The effect of this measure will be seen by these figures. In the second week of July, 1885, 1,235 deaths occurred, while the corresponding week this year shows the increased death-rate of 1,428. The mortality among children under five years was in the preceding week and year 542. This year 683. The sanitary condition of the city is, however, better this summer than last. The streets are cleaner and the number of plague spots happily few.

A. F.

*New York, September, 1886.*

